# Supplementary material for: Impact of Nutrient Intake on Body Composition in Very Low-Birth Weight Infants Following Early Progressive Enteral Feeding
Source: Nutrients. 2024 May 13;16(10):1462. doi: 10.3390/nu16101462 (PMC11124094; doi:10.3390/nu16101462)
Supplement: Supplementary file 1 [file nutrients-16-01462-s001.zip › nutrients-2989856-supplementary.pdf]

Table S1. Main differences in body composition estimates between two groups, given as median and interquartile ranges and means and standard deviations.

Characteristics	Extremely Preterm *n*=32	Very Preterm *n*=54	*p*
PMA on the day of DXA scan, weeks	36.2 (35.0–37.7)	35.4 (34.5–36.3)	**0.036**
Chronological age on the day of DXA scan, weeks	10.2 (8.2–12.3)	6.2 (4.7–7.0)	**<0.001**
Weight on the DXA day, g	2558 (± 354)	2284 (± 358)	**<0.001**
Length on the DXA day, cm	46.0 (± 1.9)	45.3 (± 2.0)	0.074
DXA estimated total mass, g	2786 (± 374)	2512 (± 371)	**0.001**
DXA estimated lean mass, g	2086 (± 246)	2012 (± 249)	0.188
DXA estimated fat mass, g	700 (± 212)	500 (± 209)	**<0.001**
DXA estimated fat mass percentage, %	24.8 (± 5.4)	19.4 (± 6.4)	**<0.001**
Sum of SFT on the day of DXA scan, mm	17.8 (±2.6)	15.9 (±2.9)	**0.004**
Sum of SFT at 12 months of CA, mm	26.9(±5.5)	26.3(±4.6)	0.661

PMA – postmenstrual age, CA – corrected age, DXA – dual X-ray absorptiometry, SFT – skinfold thickness.
